# Light People: Prof. Daoxin Dai, Dr. Patrick Lo, and Prof. Yikai Su—innovators in silicon photonics

**DOI:** 10.1038/s41377-024-01650-8

**Published:** 2024-10-11

**Authors:** Yating Wan, Chenzi Guo

**Affiliations:** 1 Electrical and Computer Engineering, the Computer, Electrical and Mathematical Sciences and Engineering Division, King Abdullah University of Science and Technology, 23955-6900 Thuwal, Saudi Arabia; 2 Changchun Institute of Optics, Fine Mechanics and Physics, Chinese Academy of Sciences, Changchun, China

**Keywords:** Integrated optics, Silicon photonics

## Abstract

In this edition of *Light People*, we are excited to feature Prof. Daoxin Dai (Zhejiang University), Prof. Yikai Su (Shanghai Jiao Tong University), and Dr. Patrick Lo (Advanced Micro Foundry Pte Ltd, Singapore), three prominent researchers shaping the future of silicon photonics. Their collaborative work addresses critical issues in silicon photonics, including reducing propagation losses, enlarging the functionalities and enhancing building blocks, integrating efficient laser sources, expanding applications, and pushing the boundaries of optical and electronic integration. Through this interview, we delve into their academic journeys, challenges, and future visions, offering insights into the ongoing evolution of silicon photonics and its potential to transform industries. For a deeper exploration of their experiences and advice, the full interview is available in the Supplementary material.

**Can you briefly describe your current work in silicon photonics, what initially attracted you to this field, and how has your focus shifted over time?**

**Yikai:** Our group, the Optical Transmission and Integrated Photonics Group, initiated research in silicon photonics in 2006, focusing primarily on silicon devices. I was attracted to this field due to silicon’s high refractive index, which facilitates high-density integration and its compatibility with existing CMOS fabrication processes. This compatibility simplifies the fabrication process when compared to materials like III–V semiconductors. Recently, our focus has broadened to include other promising materials, such as the heterogeneous integration of silicon nitride with lithium niobate, which offers unique properties that complement silicon.

**Daoxin:** My team is dedicated to developing high-performance silicon photonic devices and aiming to create large-scale silicon photonic circuits for applications like optical interconnects and optical computing. My interest began during my Ph.D. with my work on planar lightwave circuits using silica on silicon, which has weak optical confinement and limits integration density. Recognizing silicon’s potential for high-density integration owing to its strong optical confinement, my research has evolved from focusing on compact functional devices to pursuing large-scale integration of various elements on a single chip.

**Patrick:** I initially focused on hardcore CMOS technologies during my Ph.D. and initial industry work, namely on CMOS process and transistor devices research. After I moved to Singapore in 2004, I began to seek alternative semiconductor research areas, including nano-devices, shortly after in 2006, I started exploring silicon photonics, drawn by its potential in areas beyond traditional scaling limits, a concept often referred to as ‘More than Moore”, fancied just by the notion from electrons to photons and electrons. At the Institute of Microelectronics (IME) at that time, we pioneered the development of capacitor-based modulators shortly after Intel. From the very beginning, in order to be true value-adders and be differentiative from either purely academic or pure industrial research, we were very clear that our work needed to center around creating technology platforms that were transitionable to industry-nature product platforms, which subsequently catalyzed the establishment of dedicated foundries. Over time, my focus has been shifting back and forth between the essential device physics and end application, and product exploration of such attitudes has been instrumental in always guiding us in continuously commercializing our findings. With our industrial partners, the team successfully launched the first 100G coherent products and, subsequently, the 400G products. This successful transition from lab to market commercialization over the last decade has been immensely rewarding, for one, providing great confidence to both industry and academic circles. For example, you might have appreciated the trend of research funding that has been hugely poured in from private industry in addition to the typical source from the government, from a few regional activities to be very global.

**Given the rapid evolution of silicon photonics and the increased services offered by major fabs, what are the major challenges facing silicon photonics today?**

**Daoxin:** While silicon photonics has become mainstream, we still face significant challenges in achieving high-performance devices and high-yield, large-scale circuits that meet the demands of real-world applications. For instance, a typical single-mode silicon waveguide with a cross-section of 450 nm × 220 nm suffers from a propagation loss of 1–2 dB/cm, which is substantial over longer distances. Looking ahead, we need to address three areas: exploring materials beyond silicon to overcome its limitations, moving beyond the single-mode regime to reduce propagation loss and phase errors, and broadening the applications of silicon photonics with devices for visible and mid-infrared light, which holds both opportunities and challenges.

**Patrick:** From an industry perspective, expanding the applications of silicon photonics involves incorporating various materials to support different wavelengths, functionalities, and performance. Frankly, Silicon is not the best material for optics in many accounts, but it’s the best material for integration. The balance between investment and returns is critical—finding applications that are financially viable remains essential. Admittedly, although some applications have been explored, including today’s transceivers riding with the AI wave to cite an example, I have to say we have yet to discover a ‘killer application’ that would justify large-scale investment if we compare with the electronics, say CMOS alike. Close collaboration with academia is crucial as we continue searching for these high-impact applications.

**Yikai:** I agree that there’s always a balance between fundamental research and applications. From an academic viewpoint, several challenges need to be addressed. Firstly, the integration of a low-cost, high-power, and efficient laser source with silicon remains elusive. Secondly, on the modulation side, silicon is nearing its theoretical limit of ~100 GHz due to carrier dispersion effects. Surpassing this limit likely requires the incorporation of alternative materials like lithium niobate or barium titanate to reduce modulator sizes. Ultimately, heterogeneous integration may be indispensable to advance beyond the current limitations of a single material system.

**Considering the high yield rates of about 90% for advanced integrated circuits at 7** **nm or even 5** **nm, what can we expect in terms of scalability and yield for silicon photonics?**

**Partick**: In photonics, we don't need to scale down to dimensions like 5 or 3 nm because the dimensions are dictated by the wavelength. For the foreseeable future, 80 or 90 nm will likely be adequate. While some fabs, like Intel, are working with 32 nm processes on 12-inch wafers, such precision often exceeds the requirements for most photonics applications. Although these smaller dimensions are not necessary, they do help greatly achieve better uniformity in devices such as optical switches, WDM, etc. While there’s debate about the value of investing further in lithography and/or wafer size, just my personal view that for silicon photonics, the future of photonic integration lies in combining different materials and functionalities on a single chip, and sometimes for performance, assuming Si and Ge would run out of steam for bandwidth, power consumption, etc. Both heterogeneous and hybrid integration strategies have significant potential, but their success will depend on achieving the required performance at a cost that makes economic sense.

**As leading companies now deliver high-speed electronic chips to support silicon photonics, what do you perceive as the primary challenges in electro-optical integration?**

**Patrick:** The main challenge now is optimizing the integration of electronics and photonics, which often requires some performance trade-offs. Currently, some foundries tend to prioritize electronic performance, which can lead to compromises in photonic performance. A significant challenge is achieving cost-effective solutions, especially in packaging, which I believe is the biggest hurdle for adopting co-packaged optics (CPO), which requires change from the present day's supplier chain. Before the industry can commit to the best packaging solutions, it needs to recognize the tangible benefits driven by real-world applications. Again, collaboration between academia and industry is crucial to resolve these technical challenges and move forward since these new technologies require quite a broad multi-disciplinary approach, no longer confined to electronics or photonics trained.

**Given that your students have various career options ranging from academia, industry, to starting their own companies, what advice would you offer, especially considering the trend where smaller companies are often acquired by larger ones?**

**Yikai:** I typically recommend that graduates start their careers at large companies or well-known research institutes. These places offer extensive support and resources, which can help new graduates specialize and develop their skills more effectively. However, joining a startup also has its benefits, such as a broader scope of responsibilities and quicker growth opportunities in both technical knowledge and business understanding. The best choice depends on the individual’s strengths, personality, and available opportunities. There’s no one-size-fits-all answer.

**Which application do you foresee as particularly interesting for silicon photonics in the coming years?**

**Daoxin:** A promising direction is optical measurements on silicon. We aim to develop a high-performance optical spectrometer integrated into a silicon chip, which would be ultra-compact and cost-effective compared to conventional, larger systems. Although these on-chip solutions currently have limitations in resolution and spectral range, we are exploring ways to overcome these by combining high-resolution multi-channel filters with photodetector arrays and integrating them alongside electronic circuits. Another exciting area is optical computing, which is still in its early stages. With advancements in device performance and integration capabilities, optical computing could become a practical reality. However, to realize the potential of optical computing, it's crucial to first establish robust fundamental building blocks. This foundational work is essential to prevent hitting roadblocks from technical limitations.

**You mentioned building blocks. How can academia contribute to developing these, especially in passive silicon photonics?**

**Daoxin:** Passive devices form the backbone of silicon photonics systems, constituting over 80% of the components on large-scale silicon chips. While individual passive devices typically perform well, integrating them on a large scale poses challenges due to the need for extremely low loss and crosstalk across a wide bandwidth. This is challenging because silicon photonics waveguides are highly dispersive. Take a large-scale optical switch, for instance, which might include hundreds, even thousands of switching elements. Even simple structures like a 2 × 2 Mach–Zehnder switch can suffer from significant phase errors. Academia can make a significant contribution by developing devices that are highly tolerant to fabrication variations, aiming for near-perfect performance using accessible standard processes. This would minimize the need for calibration and enhance overall system reliability.

**How do foundries balance the demand for customized processes with the need to standardize in silicon photonics manufacturing?**

**Patrick:** Using the example of AMF, since its inception, standardization has been central to our strategy—it's crucial for ensuring consistent and repeatable production while minimizing the need for continuous innovation, or I shall reuse existing proven innovation. However, the dynamic nature of new applications and evolving technologies often requires a degree of customization. Flexibility and a willingness to adapt are essential for creating new products and supporting both industry and academia, which eventually lead to standardization with the field experienced so-called best-known-method in statistical sense. Photonics, unlike CMOS, requires unique optimization at every stage, making customization indispensable. Yet, it's important to recognize that standardization is not just about simple uniformization of practices in devices or processes; it's an attitude and a methodology. Balancing customization means focusing on manufacturability, ensuring that designs can be sustained and can scale up for mass production. It's also important to design for variability and testability. For example, in photonics manufacturing, the first layer—the passive circuit—dictates all subsequent optical functionalities, meaning any customization needs to work within this core structure.

**How competitive is silicon photonics with technologies like VCSEL, indium phosphide for single-mode applications, and lithium niobate for future coherent applications?**

**Yikai:** Each material platform has its strengths and weaknesses depending on the application. Silicon photonics excels in high-density integration and is versatile enough to allow for hybrid or heterogeneous integration with other materials. III–V materials, such as indium phosphide, are effective in VCSELs or EMLs, offering low power consumption per bit, and are well-suited for optical interconnects. Lithium niobate stands out for its high-speed modulation capabilities, achieving speeds up to terahertz, making it ideal for high-data-rate applications like 400G and beyond. The choice between these materials often hinges on specific application needs and practical considerations like cost and production yield.

**Daoxin:** Silicon photonics is particularly cost-effective and suitable for large-scale integration, making it a strong candidate for consumer electronics. For long-haul networks, where performance is crucial, integrating materials like lithium niobate is necessary for ultra-fast optical modulation. However, lithium niobate alone does not offer a really high electro-optic (EO) coefficient, prompting interest in materials like barium titanate (BTO) for creating ultra-compact optical modulators. As we continue exploring new materials to enhance performance, it becomes clear that combining multiple materials—often with silicon as the foundation—offers a promising path for future technological advancements.

**You mentioned exploring more materials in the future. What potential materials are you referring to?**

**Daoxin:** Currently, there's interest in integrating zero-dimensional, one-dimensional, and two-dimensional materials, each offering unique properties. These materials are not only cost-effective to fabricate but also versatile. For instance, you can place 2D materials directly on a wafer or on top of a waveguide to create modulators or detectors. However, this technology is still in its early stages, and substantial work is needed, especially in achieving wafer-scale integration.

**Given the competition between technologies like the 8** **×** **200** **Gbps solution from EML and silicon photonics in the data center, what’s your perspective from an industry standpoint?**

**Patrick:** The competition between technologies like EML and silicon photonics isn't just about technical performance; it crucially involves cost, supply chain maturity, and product lead times. Silicon photonics typically involves more layers, resulting in longer production cycles and a less mature supply chain so far compared to III–V technologies. Packaging limitations also place silicon photonics at a disadvantage in direct competition with EML. However, silicon photonics offers advantages in high-level integration scenarios. For instance, while EML remains slightly cheaper for 400G DR or FR solutions, silicon photonics is more cost-effective for 400G ZR modules. At 1.6 T, where higher integration and more lasers are needed, silicon photonics demonstrates a clear cost benefit. Looking ahead, I expect increased adoption of silicon photonics in 1.6 T modules, primarily driven by cost advantages. Companies that leverage both silicon photonics and III–V technologies are best positioned to indicate if and when silicon photonics becomes more cost-effective. Ultimately, while both technologies aim to reduce costs and enhance performance, silicon photonics is likely to secure a cost advantage at higher capacities like 1.6 T and beyond.

**Some argue that all components should be made from a single material instead of integrating different materials. What’s your vision about this?**

**Daoxin:** Integrating different materials remains necessary because no single material system can fulfill all functions. III–V semiconductors, for example, are excellent for active devices but have limitations like smaller wafer sizes and higher optical losses. That’s why integrating III–V on silicon has been popular. However, for applications where a single or few lasers are needed, an off-chip approach might be suitable. For larger laser arrays, heterogeneous integration becomes necessary to manage coupling efficiently. For materials like lithium niobate, issues such as weak optical confinement and sidewall angles can introduce large sizes and undesired mode conversions. Alternatives like PZT, which can be spin-coated on silicon, may offer low-cost, efficient alternatives for certain applications. Our strategy should aim to minimize the number of different materials while still leveraging multiple platforms where necessary to balance cost-effectiveness and performance.

**Yikai:** I agree that while lithium niobate excels in high-speed operations, its fabrication complexities, such as managing the sidewall angle for large-scale integration, present significant challenges. The diversity of applications requires a mix of materials since no single material system offers a solution for all functionalities. For instance, while III–V materials are excellent for active components, they suffer from complex layering and low-yield issues. Therefore, the future will likely require the co-integration of multiple materials on unified platforms to meet high technical standards.

**Daoxin:** One point to add, lithium niobate’s anisotropic nature also complicates device design compared to more straightforward silicon photonic waveguides. However, its unique nonlinear properties offer substantial advantages, contributing to its continued relevance in specific applications.

**Patrick:** As a CTO of an industry organization, in addition to looking after the existing product and market space, it’s crucial to be forward-thinking and open to all potential technological avenues years ahead, ensuring we don’t miss out on emerging opportunities while also maximizing our current capabilities. For instance, one area we’re exploring with our collaborators is the use of polymer-based modulators. Polymers, compared to Lithium Niobate, have a better electro-optic coefficient, achieving as low as 0.5 V-cm, with bandwidth largely influenced by the electrical field. This makes polymers an appealing option since they don’t require additional tools or significant investment for integration into our existing processes. We remain cautious about introducing newer materials like lithium niobate into full production until they demonstrate the necessary reliability, maturity, and cost-effectiveness. Our material choices are guided by both technical and economic factors, including the specific requirements of the product, whether it be coherent-based or IM-DD-based. It's an exciting time in the field, with many good options to explore. Each technology will find its role over time, and it’s not about one platform immediately replacing another. These discussions are important as they drive innovation and keep us focused on what we can achieve and apply, rather than be discouraged by the limitations alone. Challenges and Opportunities always go hand-in-hand.

**Throughout your career, what has been the most rewarding part?**

**Yikai:** Several aspects of my career have been particularly rewarding. First, I’ve always been passionate about my research. It’s been exciting to see the field grow with new researchers and breakthroughs. It’s also fulfilling to see our research translate into commercial products like silicon coherent receivers and transmitters. Looking ahead, I’m excited about the future possibilities for photonic chips and their commercialization over the next decade.

**Daoxin:** For me, the most rewarding aspect has been developing a library of high-performance building blocks and advancing large-scale silicon photonic systems on a chip. In the future, we aim to reduce losses further, enhance optical power from laser sources, and improve detection sensitivity to boost the signal-to-noise ratio. We’re also focused on achieving high uniformity at the wafer scale for extensive integration and exploring new applications in silicon photonics beyond the traditional 1550 nm wavelength band.

**Patrick:** What I find most rewarding is seeing our technology mature into commercial products. After graduation, I was attracted to the industry because it offered the chance to bridge technology development and commercial success. Today, with technology cycles getting shorter, I’m motivated by the challenge of quickly bringing innovations to market and seeing their impact. I often remind academic partners of the importance of sharing detailed reports on engineering efforts—not just the successes, but also the failures—as these insights are valuable for product development in industry. Ultimately, products generate revenue, which fuels further investment to trigger research initiatives.

**What is the top challenge you would like to solve for the future of silicon photonics?**

**Yikai:** The main challenge I’m focusing on is achieving high-capacity photonic chips. There’s a DARPA project targeting 1 petabit/s for I/O chips by 2030, which has highlighted several key challenges in photonic integration, not just in silicon photonics. First, the chip needed to reach 1 petabit/s with channel counts ranging from 1000 to 10,000. This requires a dramatic reduction in component size, potentially through entirely new technologies. Secondly, improving power efficiency is crucial, particularly for modulators, detectors, and laser sources. Thirdly, to manage such high capacities with minimal fibers, we’d need to greatly expand bandwidth capabilities, using advanced multiplexing technologies like space, mode, or wavelength division multiplexing. Additionally, a new DARPA program is exploring 3D photonic integrated chips, which could involve creating multi-layered photonic structures within and between chips. If achieving petabit capacity on a single chip proves too challenging, the alternative would be to distribute this capacity across multiple interconnected chips. Addressing these challenges demands the invention of new technologies and revisiting fundamental theories, as we are approaching theoretical limits. The next decade promises significant opportunities for pioneering research and innovation in this field.

**What do you think the industry needs most from academia?**

**Patrick:** Students! We need well-rounded students trained in diverse fields including thermal management, mechanical reliability, and not just optics and electronics. Optics is a complex field influenced by numerous factors and requires a distinct approach. To foster innovation and address the challenges of modern technology, academia and industry must collaborate closely. Academia should strive to push the boundaries of what’s possible, without being constrained by current supply chain limitations. It’s through tackling these complex needs that true innovation can emerge.

**What insights do you expect Light: Science & Applications to bring?**

**Patrick:** Throughout my career, I’ve found the most value in understanding the methodologies and challenges described in technical papers, rather than just the final results. Documenting failures and setbacks is crucial—it helps the scientific community learn from past mistakes instead of merely celebrating significant achievements. I encourage sharing not only successes but also the practical difficulties encountered along the way. This approach can foster a richer, more beneficial learning environment for everyone.

**Yikai:** We’ve published several papers in *LSA*, including a notable collaboration on a monolithic silicon laser with the Institute of Physics. Such interdisciplinary research is crucial for breakthroughs. I’d like to see challenging and important topics requesting multi-field knowledge.

**Daoxin:** I’m eager to see the journal address some of the biggest challenges in the field, particularly those innovative ideas from young researchers who are shaping the future of this field.

**Figure:**
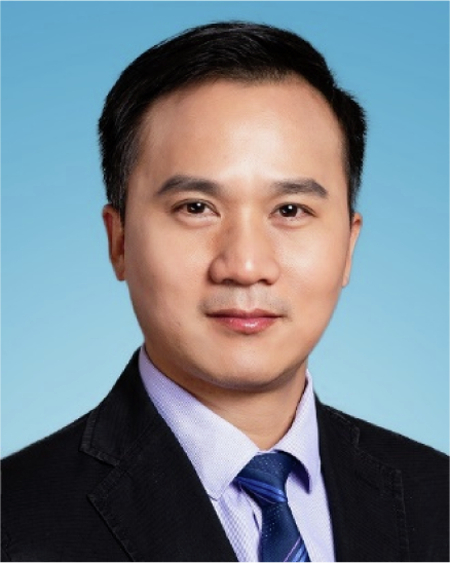
**Prof. Daoxin Dai** received a B.Eng. degree from Zhejiang University (ZJU), China, and a Ph.D. degree from the Royal Institute of Technology, Stockholm, Sweden, in 2000 and 2005, respectively. He joined Zhejiang University as an assistant professor in 2005 and became an associate professor in 2007 and a full professor in 2011. He worked at the University of California at Santa Barbara (UCSB) as a visiting scholar during the years of 2008–2011. Currently, Prof. Daoxin Dai is the Qiushi Distinguished Professor of ZJU and is leading the silicon-integrated nanophotonics group at ZJU. He has been working on integrated photonics since 2000, particularly including multimode silicon photonics as well as silicon-plus photonics. Prof. Dai has published more than 320 refereed international journals papers in Science, Nature, Nature Photonics, Light: Science and Applications, Optics Letters, etc. He has been invited to give >100 plenary/tutorial/keynote/invited talks at prestigious national/international conferences, including OFC 2016/2021. Prof. Daoxin Dai is one of the Most Cited Chinese Researchers in 2014-2023 (from Elsevier), the Winner of the National Science Fund for Distinguished Young Scholars (2017) and the Wang-Daheng Award of Optics (2020). He was elected as an Optica (former OSA) Fellow in 2021.

**Figure:**
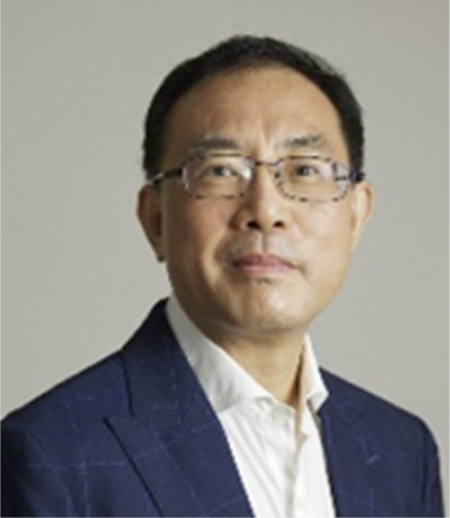
**Dr. Patrick Lo** received Ph.D. in electrical and computer engineering from the University of Texas at Austin, USA, in 1992. He was with Integrated Device Technology, Inc., USA, on Silicon-CMOS semiconductor manufacturing areas in process and integration R&D. From 2004 to 2017, he was with IME/Singapore, where he was the Laboratory Director and Program Directors of Nanoelectronics and Photonics program, subsequently the Deputy Executive Director of the institute covering R&D. At 2017, he co-founded the company as Advanced Micro Foundry Pte Ltd, being the President and now as CTO, focusing on silicon photonics manufacturing technology industrialization and commercialization, with dedicated Si Photonics Foundry Services and Solutions. He was the recipient of the IEEE George E. Smith Award for Best Paper, Published in the IEEE Electronic Device Letters in 2007. He has won both Singapore’s National Technology Award and President Technology Award for his works on nano-Electronics and Photonics in 2008 and 2010, respectively

**Figure:**
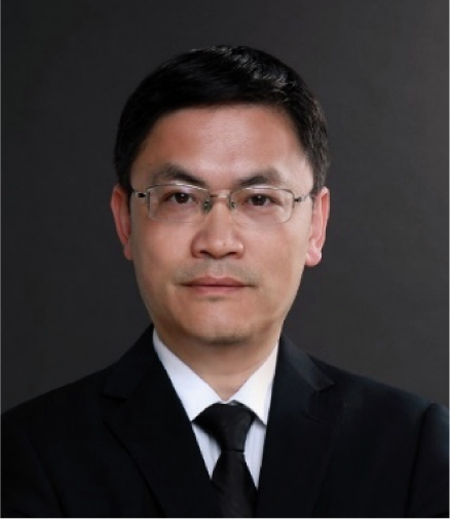
**Prof. Yikai Su** received a Ph.D. degree in EE from Northwestern University, Illinois, USA, in 2001. Subsequently, he worked at Crawford Hill Laboratory of Bell Laboratories in Holmdel, NJ. He joined Shanghai Jiao Tong University in 2004, where he is currently a full professor leading a group to work on optical transmission and integrated photonics. He was also the founding director of a university-level fabrication facility (center for advanced electronic materials and devices). His research areas cover silicon photonic devices, heterogeneous integration, optical transmission, and switching. He has over 500 publications in international journals and conferences and gave >80 invited talks at conferences, including OFC and CLEO. Prof. Su is an advisory board member for ACS Photonics and APL Photonics. He also served as a subcommittee chair of OFC D2 2023, a general co-chair of ACP 2021 and OECC 2023, and a TPC member of many international conferences, including OFC, ECOC, CLEO, and IPC. Prof. Su is a Fellow of Optica

## Supplementary information

Supplementary Video-Full Interview

